# Outcomes of infants born to pregnant women with syphilis: a nationwide study in Korea

**DOI:** 10.1186/s12887-021-02502-9

**Published:** 2021-01-22

**Authors:** Joohee Lim, So Jin Yoon, Jeong Eun Shin, Jung Ho Han, Soon Min Lee, Ho Seon Eun, Min Soo Park, Kook In Park

**Affiliations:** Department of Pediatrics, Yonsei University College of Medicine, Yonsei-ro 50, Seodaemun-gu, 03722 Seoul, Republic of Korea

**Keywords:** Congenital syphilis, Neurosyphilis, Outcome

## Abstract

**Background:**

Despite the expansion of antenatal syphilis screening programs, congenital syphilis (CS) remains a concern.

**Purpose:**

This study aimed to analyze the manifestation and progress of CS, including treatment and follow-up, based on a nationwide study.

**Methods:**

From the Korean National Health Insurance Service database, a total of 548 infants were examined for CS during their first year of life from 2013 to 2018. Neurosyphilis and complications were investigated using the International Classification of Diseases-10 codes.

**Results:**

The birth rate of infants from mothers with syphilis was 2.8 per 10,000 live births for 5 years, which is not indicative of a decreasing trend. Overall, 148 infants were proven or highly probable or possible of having CS with treatment for 10 days; 66 infants were possible or less likely of having CS with only 1-day treatment. Jaundice (56 %) was common, followed by hearing impairment (14 %), renal disease (8 %), and mental retardation (8 %). Fourteen cases of neurosyphilis occurred. Infants with complications, including mental retardation, eye involvement, hearing impairment, or renal disease, were significantly associated with neurosyphilis (OR 8.49, *P* < 0.0001). Of 250 patients who received treatment, 92.8 % were treated with one medication: benzathine penicillin was used in 73 % of patients. Only four patients were re-treated due to treatment failure. In addition to the treponemal test, fluorescent treponemal antibody-absorption was the most utilized tool for diagnosis and follow-up.

**Conclusions:**

Establishing standardized guidelines for the evaluation of CS, as well as the establishment of treatment regimens and follow up-plans for the disease, at a national level would help improve maternal and neonatal care and facilitate the eradication of CS in Korea.

## Background

Congenital syphilis (CS), caused by *Treponema pallidum*, can be passed from the mother to infant in utero or at birth by exposure to infected maternal lesions. CS is a cause of considerable morbidity and mortality worldwide [1]. Syphilis is generally thought to be eradicated, although it still remains in both developing and developed countries [2, 3]. The World Health Organization (WHO) estimates that approximately one million pregnant women around the world are infected with syphilis each year [4]. Meanwhile, in Korea, the sentinel surveillance system, which started in 2001, has recorded increases in the number of CS cases. A total of 94 cases were reported between 2001 and 2010, while 99 cases were reported from 2011 to 2014 [5]. According to data from the Korea Centers for Disease Control and Prevention, the rates of syphilis and congenital syphilis in 2014 were 1.42 cases per 100,000 people and 0.05 cases per 100,000 [6].

Untreated syphilis during pregnancy is transmitted in > 70 % of affected infants, and fetal or perinatal death occurs in 40 %. Unfortunately, more than two-thirds of live neonates born to untreated or inadequately treated mothers does not present any symptom at first. However, there are few national reports on infants with CS. A study in Louisiana and Florida from 2013 to 2014 showed that 20.3 % of a total of 3,497 syphilis cases were found in pregnant women, and mother-to-child transmission occurred in 22 %, leading to five stillbirths and five deaths after birth due to CS. Thus, for every 4.6 pregnant women with syphilis, there was one case of CS. Therefore, all neonates born to mothers with reactive nontreponemal/treponemal test results are recommended to do a quantitative nontreponemal test using neonate’s serum and be considered for proper treatment.

Treatment should be decided on the basis of identification of syphilis in the mother; adequacy of maternal treatment; presence of clinical, laboratory, or radiologic evidence of syphilis in neonate; and comparison of maternal and neonatal serologic titers. Clinical symptoms are divided into early signs which appears in the first 2 years of life and late signs developing later over the first two decades of life [7]. Symptoms such as hearing loss, hydrocephalus, optic nerve atrophy, and mental retardation require follow-up for at least 2 years [8]. Renal involvements may vary from simple albuminuria to significant nephritis or nephrotic syndrome [7, 9]. Physicians should be aware of the various clinical features of syphilis to enable early diagnosis. However, data on the clinical manifestations and long-term outcomes of CS are limited.

Although CS is a preventable disease, its incidence has not decreased in the last 10 years. As understanding of the neonatal outcomes of pregnant women with syphilis could help with deciding on the appropriate treatment, this study sought to analyze the manifestations and progression of CS, including treatment and follow-up.

## Methods

The Korean National Health Insurance Service (NHIS) stores healthcare data, such as diagnostic codes, diagnostic tests, procedures, and prescription medications, for Korean residents. Nearly 98 % of Korean residents are covered by the NHIS and 2 % are covered by medical aid [10]. NHIS records also include demographic data of the beneficiaries, such as age, sex, residential area, and income status [11]. From the NHIS-NSC data for 2014–2018, we selected infants under 1 year of age who underwent nontreponemal and/or treponemal tests and were diagnosed with CS (International Classification of Diseases-10 codes: A50.0, A50.1, A50.2, A50.9). Finally, a total of 548 infants were followed up from the date of testing for CS to December 31, 2018. We used birth statistics to estimate the prevalence of CS based on the number of live births [12]. The International Classification of Diseases-10 codes were used to determine the infants with clinical manifestations or complications, including jaundice, hepatosplenomegaly, ascites, renal disease, optic nerve atopy, hearing impairment, mental retardation, intrauterine growth retardation, and hydrops fetalis. Neurosyphilis with positive cerebrospinal fluid VDRL was confirmed for infants diagnosed with the International Classification of Diseases-10 codes A52.1, A52.2, A52.3, and A50.4. Benzathine penicillin or aqueous penicillin was used for treatment.

### Statistical analyses

The cohort was stratified by the study year. One-way ANOVA and t tests were used to compare the neonatal characteristics and complications among different groups. We analyzed the data by using SAS version 9.4 (SAS Institute, Cary, North Carolina). *P* values < 0.05 were considered statistically significant.

### Ethics statement

In this work, all identifiable variables, including claim-, individual-, and organizational-level identification numbers, were re-generated randomly and anonymized before its use to protect patient’s privacy. This study used NHIS data (NHIS-2020-1-100) made by NHIS. We obtained adequate administrative permissions of NHIS to use the data for research purposes. We ensure the security of the data and confidentiality of the information contained in the data. The study protocol was approved by the Institutional Review Board (IRB) of Gangnam Severance Hospital (IRB No. 3-2019-0147).

## Results

**Fig. 1:**
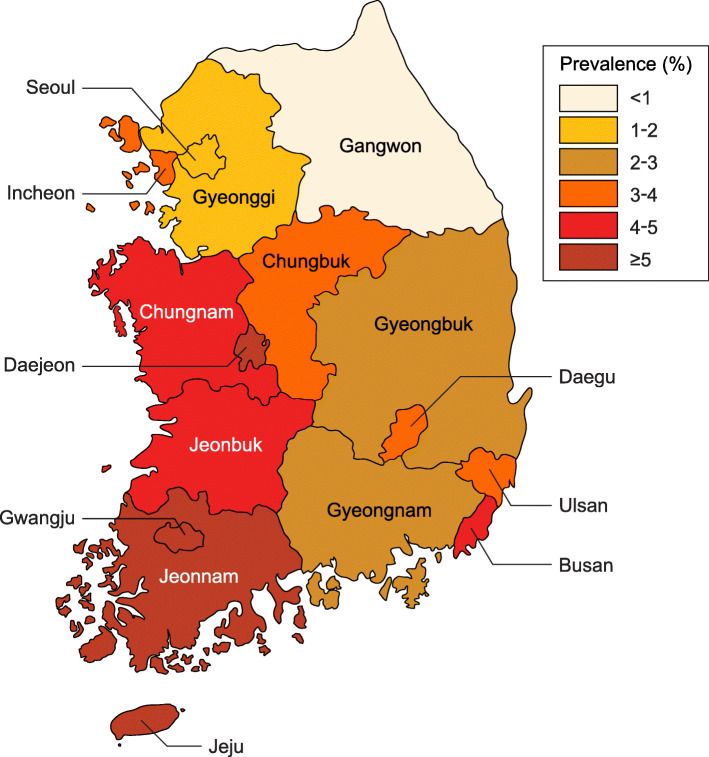
Prevalence of infants born to mothers with syphilis by region (/10,000 births). This figure was designed by medical illustration & design of medical research support section, Yonsei University College of Medicine, Seoul, Korea

A total of 548 infants born between 2014 and 2018 were evaluated with a nontreponemal test for the diagnosis of CS. Reflective of a steady rate, the birth rate of infants from mothers with syphilis was 2.8 per 10,000 live births during the 5-year period. The preterm birth rate was 0.5 per 10,000 live births, and the term birth rate was 3.0 per 10,000 live births. Prevalence distribution according to province is shown in Fig. 1. Seoul and its surrounding areas showed a lower incidence of CS compared to other regions.

Among 548 infants, CS was considered unlikely in 298 infants. Overall, 148 infants who were proven or considered highly probable or possible of having CS were treated for 10 days. Meanwhile, 66 infants with possible or less probable CS were treated for only 1 day with benzamine penicillin. Males accounted for 53.4 % of all cases.

Jaundice was the most common symptom (56 %), followed by hearing impairment (14 %), renal disease (8 %), and mental retardation (8 %). Small for gestational age or intrauterine growth restriction was observed in 6 % of cases (Table 1). A total of 14 cases of neurosyphilis occurred over the 5-year study period. Among neurosyphilis patients, mental retardation occurred in one patient, and hearing impairment occurred in six patients. Hearing impairment was more frequent in patients with neurosyphilis than in those without (*P* < 0.0001). Complications including mental retardation, eye involvement, hearing impairment, and renal disease were significantly associated with neurosyphilis (OR, 8.49; 95 % CI, 2.7–26.6; *P* < 0.0001). Infants treated with aqueous penicillin showed a higher risk of complications compared to those treated with benzathine penicillin (OR, 2.42; 95 % CI, 1.1–6.2; *P* < 0.0001). Syphilis combined with noted complications tended to increase the risk of treatment failure, which resulted in prolonged treatment duration (OR, 1.09; 95 % CI, 1.03–1.15; *p* = 0.0005; Table 2). Sex, preterm birth, and birth year did not significantly affect the rate of complications.

**Table 1:** Clinical characteristics of infants evaluated for congenital syphilis

	2014 *N* = 77	2015 *N* = 56	2016 *N* = 45	2017 *N* = 32	2018 *N* = 40	Total *N* = 250
Prevalence /10,000 births	3.86	2.78	2.42	1.96	2.78	2.80
Male, n (%)	39 (50)	32 (57)	23(51)	16 (49)	24 (61)	134 (54)
Preterm, n (%)	3 (4)	1 (2)	0 (0)	0 (0)	1 (3)	5 (1)
Jaundice, n (%)	48 (62)	31 (58)	23 (51)	14 (45)	24 (59)	140 (56)
Hepatosplenomegaly, n (%)	2 (3)	1 (2)	0 (0)	0 (0)	1 (3)	4 (1)
Ascites, n (%)	2 (3)	1 (2)	1 (2)	0 (0)	1 (3)	5 (2)
Renal disease, n (%)	7 (9)	5 (9)	5 (11)	2 (6)	2 (5)	21 (8)
Optic nerve atrophy, n (%)	0 (0)	0 (0)	1 (2)	0 (0)	0 (0)	1 (0)
Hearing impairment, n (%)	12 (16)	8 (14)	7 (16)	6 (19)	1 (3)	34 (14)
Mental retardation, n (%)	8 (10)	7 (13)	4 (9)	0 (0)	0 (0)	19 (8)
IUGR or SGA, n (%)	4 (5)	3 (5)	3 (7)	3 (9)	2 (5)	15 (6)
Hydrops fetalis, n (%)	1 (1)	0 (0)	0 (0)	0 (0)	0 (0)	1(0)

*IUGR* intrauterine growth restriction, *SGA* small for gestational age

**Table 2:** Comparisons of characteristics between infants with and without complications of congenital syphilis

Year	MR + Eye + Hearing + Renal (*N* = 51)	None (*N* = 199)	*P*-value
2014	16 (21 %)	60 (79 %)	0.4626
2015	15 (22 %)	53 (78 %)	
2016	9 (21 %)	34 (79 %)	
2017	8 (25 %)	24 (75 %)	
2018	3 (3 %)	32 (91 %)	
Male	21 (41 %)	108 (53 %)	0.1248
Preterm	0 (0 %)	7 (3 %)	0.6155
Neurosyphilis	7 (14 %)	7 (3 %)	< 0.0001
Aqueous penicillin	7 (14 %)	57 (28 %)	0.0394
Benzathine penicillin	39 (76 %)	131 (63 %)	
Duration of Tx ≥ 10	48 (6 %)	165 (17 %)	0.1691

*MR* mental retardation, *Tx* treatment

Among 250 patients who underwent treatment, 92.8 % were treated with one medication (aqueous crystalline penicillin G or benzathine penicillin G); benzathine penicillin was used in 73 % of patients. Eighteen patients received both benzathine penicillin G and aqueous crystalline penicillin G (Table 3). Only four patients were re-treated due to treatment failure: one each at 2 weeks, 1 month, 3 months, and 6 months. Two received aqueous crystalline penicillin G, and the other two received both aqueous crystalline penicillin G and benzathine penicillin G, with an average treatment duration or median duration (Table 3 shows median treatment durations). There were no sex disparities therein, and all births were full term. The re-treatment group comprised one infant with renal involvement and one infant with hearing impairment. The treatment durations for the initial treatment were 1 day in one infant and 10 and 16 days, respectively, in the other two infants.

**Table 3:** Treatment drugs and duration

Initial treatment N = 250	Treatment with one drug, n (%)	232 (93 %)
	Aqueous crystalline penicillin G, n (%)	80 (32 %)
	Benzathine penicillin G, n (%)	182 (73 %)
	Duration of treatment 1 day, n (%)	66 (26 %)
	2–9 days, n (%)	36 (14 %)
	≥10 days, n (%)	148 (59 %)
Re-treatment N = 4	Treatment with one drug, n (%)	2 (50 %)
	Aqueous crystalline penicillin G, n (%)	2 (50 %)
	Benzathine penicillin G, n (%)	4 (100 %)
	Duration of treatment, median (range)	15.5 (3–26)

The numbers and methods of treponemal tests for follow-up in 2018 are described in Table 4. In addition to the nontreponemal test, fluorescent treponemal antibody-absorption (FTA-ABS) was the most utilized tool for diagnosis during follow-up; treponemal pallidum particle agglutination was the second most commonly used method.

**Table 4:** Numbers and methods of treponemal tests additionally performed with nontreponemal tests for follow-up in 2018

		At birth *N* = 60	1st follow-up *N* = 11	2nd follow-up *N* = 5	3rd follow-up *N* = 1
Number of tests	1	38 (63 %)	5 (45 %)	3 (60 %)	0 (0 %)
	2	15 (25 %)	4 (36 %)	1 (20 %)	1 (100 %)
	3	7 (12 %)	1 (9 %)	1 (20 %)	0 (0 %)
	4	0 (0 %)	1 (9 %)	0 (0 %)	0 (0 %)
Test method	TPPA	20 (33 %)	8 (73 %)	3 (60 %)	0 (0 %)
	EIA	20 (33 %)	5 (45 %)	4 (80 %)	1 (100 %)
	FTA-ABS	48 (80 %)	6 (55 %)	1 (20 %)	1 (100 %)
	PCR	1 (2 %)	0 (0 %)	3 (60 %)	0 (0 %)

*TPPA* treponema pallidum particle agglutination, *EIA* enzyme immunoassay, *FTA-ABS* fluorescent treponemal antibody absorbed test, *PCR* polymerase chain reaction

## Discussion

Despite the expansion of antenatal syphilis screening programs over the past few decades, syphilis continues to be a major public health concern worldwide. This is the first nationwide study on the manifestation and treatment of CS in Korea. Given the chances of transplacental transmission during pregnancy at any stage of maternal disease [13], the WHO launched a global initiative against mother-to-child transmission of syphilis in 2007, by promoting antenatal screenings in the first and third trimesters in all pregnant woman. Currently in Korea, pregnant women are routinely tested for syphilis at the beginning of pregnancy, and those who are at high risk of syphilis are advised to take an additional test in the third trimester. However, in recent years, CS has re-emerged in both developing and higher income countries, including the United States and Canada [14, 15].

Syphilis in Korean adults decreased from 2.5 % in 1977 to 0.2 % in 2000. The number of CS cases declined between 2008 and 2012, followed by another sharp increase from 2012 to 2014, with an increase from 8.4 to 11.6 cases per 100,000 live births [16]. Based on our data over the last 5 years, we noted that while the number of syphilis patients has tended to decrease, the prevalence of CS has fluctuated, ranging between 1.4 to 3.8 % for the past 5 years, which could be attributed to the increasing prevalence of marriages between Koreans and non-Koreans [5].

Seoul and its surrounding areas showed a lower incidence of syphilis compared to other regions. The disease prevalence tended to be removed with further distance from the capital and surrounding areas. This could be related to variations in antenatal care systems and patient compliance to antenatal care. Indeed, a higher incidence of CS in immigrant mothers has been reported due to failure of prenatal care [5]. Also, a higher incidence of gestational syphilis was observed in women of lower socioeconomic status and women with lower education [17, 18]. In this study, socioeconomic data were not included, and only trends by region could be seen.

Intrauterine infection can result in spontaneous abortion, hydrops fetalis, preterm birth, and low birth weight. Clinical manifestations in infected infants within the first 1–2 months of age include hepatosplenomegaly, lymphadenopathy, rash, mucocutaneous lesions, copious nasal secretions, pneumonia, hemolytic anemia, thrombocytopenia, and skeletal involvement [19]. Due to the limited maternal information in the current study, syphilis-related stillbirths/abortions were not included. Only one case of hydrops fetalis was noted, and the prevalence of preterm births was 0.495 per 10,000 births, compared to 2.976 per 10,000 births for term births. There was no case of mortality after birth. Over the last 5 years, male patients showed a non-significantly higher prevalence (53 %), similar to temporal studies conducted in the United Kingdom and Brazil [20, 21].

The surveillance of neurosyphilis, an uncommon but severe consequence of syphilis, is complex [22, 23]. Out of 14 patients (2.5 %) with neurosyphilis, mental retardation occurred in one case, and hearing impairment occurred in six patients (43 %). As early diagnosis and treatment are important to prevent late manifestations of the infection, we wish to emphasize the importance of screening for neurosyphilis in asymptomatic patients, even if symptoms are lacking for a diagnosis of syphilis, as the central nervous system is crucial for neurodevelopmental outcomes.

While the prognosis is considered to be very good for infected infants treated during the first 2 months of life, if left untreated, disease progression may lead to death or disability in children [24]. In our study, no death was noted; however, over 20 % of the subjects suffered from complications such as mental retardation, eye and renal involvement, and hearing impairment. The presence of complications led to a prolonged duration of treatment. Complication rates were similar throughout the 5 years, except in 2018, potentially reflecting the timepoint of data collection. Patients who received aqueous penicillin G had more complications and neurosyphilis compared to those who received benzathine penicillin G. Sex and preterm infants were not significantly associated with complication risk. In addition to high-quality antenatal screening and care, early detection of neurosyphilis and appropriate treatment indications with benzathine penicillin G can also improve the prognosis.

The recommended duration of treatment for definite or probable CS is intravenous penicillin G for 10 days. If more than 1 day of therapy is missed, the whole course must be started again. In infants with possible syphilis, a single intramuscular dose of benzathine penicillin is an alternative treatment choice in select circumstances, but only if follow-up is assured [25]. According to our data, benzathine penicillin G is prescribed more frequently than aqueous crystalline penicillin G, with a variable treatment duration. Four patients underwent re-treatment with various manifestations, treatment regimens, and durations, which may be due to the rare prevalence of the disease and site differences. This reflects the lack of standard guidelines for evaluation and therapeutic measures of CS.

Once CS is diagnosed, serial laboratory follow-up is required to assess the duration of treatment [26]. In this study, the number of tests during serial follow-up varied from one to four. Testing methods also varied. Reverse sequence testing is emerging as a high throughput and cost-effective method for screening syphilis [27]; however, it is still limited in the clinical setting. Meanwhile, out data have shown different algorithms of follow-up tests between sites. Considering the scarce prevalence of CS, it is important to share a standardized algorithm for the evaluation and treatment of CS at the national level to improve treatment outcomes.

In Korea, cases of CS have been reported occasionally in specific case reports. There are no published data with yearly long-term follow-up. Differences in the numbers reported in the Centers for Disease Control (10–33 infants with proven CS) and NHIS databases indicate the clinical complexity of diagnosis and limitations of self-reporting. A strength of our study was that it used nationwide accumulative data with updates on recent rates of CS, including long-term complications.

A limitation of this study was that it depended on infant claim data; therefore, maternal information, including adequate treatment, spontaneous abortion, and stillbirths, were not included. Data analysis after 2018 can add some additional trends and outcomes of CS. The data did not have records collected from laboratories, notably on the severity of conditions and health behavior of beneficiaries. Since the information was obtained from the diagnosis code entered by each hospital, there could be data omission or limited detailed information about the diseases of each subject. There could be a discrepancy between the diagnoses entered in the database and the actual diseases that the patients had. Furthermore, as the claims data were generated to reimburse healthcare services eligible for coverage, non-covered healthcare services were not assessed. Information about the residence of beneficiaries may not be reliable, since HIRA data are collected based on the location of providers; therefore, a beneficiary may have received healthcare services in a different area from where they actually reside.

Another limitation of our study was that it only included infants born in Korea, and the results may not be generalizable to other regions in the world.

## Conclusions

In conclusion, although antenatal syphilis screening programs have expanded, CS still remains a problem in Korea. Due to the rarity of CS, sharing standardized guidelines for evaluating CS as well as establishing treatment regimens and follow-up plans at a national level are required to improve the quality of maternal and neonatal care, which could lead to the eradication of CS.

## Data Availability

There are ethical restrictions on sharing an identified dataset, unless permitted by the Korean National Health Insurance Service. Data availability was subjected to the Act on Bioethics and Safety [Law No. 1518, article 18 (Provision of Personal Information)]. Contact for sharing or accessing the data can be possible only through the data committee of Korean National Health Insurance Service (http://nhiss.or.kr) and after gaining permission from the Korean National Health Insurance Service.

## Abbreviations

CS: Congenital syphilis
WHO: World Health Organization
NHIS: Korean National Health Insurance Service
IRB: Institutional Review Board
FTA-ABS: Fluorescent treponemal antibody-absorption
